# ‘They just walk away’ – women’s perception of being silenced by antenatal health workers: a qualitative study on women survivors of domestic violence in Nepal

**DOI:** 10.3402/gha.v9.31838

**Published:** 2016-12-14

**Authors:** Poonam Rishal, Sunil Kumar Joshi, Mirjam Lukasse, Berit Schei, Katarina Swahnberg, on behalf of ADVANCE Study Group

**Affiliations:** 1 Department of Public Health and General Practice, Faculty of Medicine, Norwegian University of Science and Technology (NTNU), Trondheim, Norway; 2 Department of Community Medicine, Kathmandu Medical College, Kathmandu, Nepal; 3 Faculty of Health Sciences, Department of Nursing and Health Promotion, Oslo and Akershus University College of Applied Sciences, Oslo, Norway; 4 Department of Obstetrics and Gynecology, St. Olavs Hospital, Trondheim University Hospital, Trondheim, Norway; 5 Department of Health and Caring Sciences, Faculty of Health and Life Science, Linnaeus University, Kalmar, Sweden

**Keywords:** domestic violence, qualitative research, quality of care, help seeking, abuse in health care

## Abstract

**Background:**

Domestic violence during pregnancy has detrimental effects on the health of the mother and the newborn. Antenatal care provides a ‘window of opportunity’ to identify and assist victims of domestic violence during pregnancy. Little is known about the experience, needs, and expectations from the women's perspective in relation to domestic violence in Nepal.

**Objective:**

Our study aims to explore how women who have experienced domestic violence evaluate their antenatal care and their expectations and needs from health centers.

**Design:**

Twelve in-depth interviews were conducted among women who had experienced domestic violence during pregnancy and utilized antenatal care. The women were recruited from two different organizations in Nepal.

**Results:**

Women in our study concealed their experience of domestic violence due to fear of being insulted, discriminated, and negative attitudes of the health care providers. The women wished that the health care providers were compassionate and asked them about their experience, ensured confidentiality and privacy, and referred them to services that is free of cost.

**Conclusions:**

Findings from our study may help the health care providers to change their attitudes toward women survivors of domestic violence. Identifying and assisting these women through antenatal care could result in improved services for them and their newborns.

## Introduction

The United Nation's (UN) Declaration on the Elimination of Violence against Women has defined domestic violence (DV) as physical, psychological, and sexual violence that occurs within a private sphere, generally between individuals who are related through blood or intimacy (1). In 2009, the Ministry of Law and Justice in Nepal defined DV as ‘any physical, mental, sexual, or economic harm perpetrated by one person on another with whom he or she has a family relationship, including acts of reprimand or emotional harm’ (2).

Globally, 13–61% of women between 15 and 49 years of age experience violence perpetrated by their intimate partners (3). The Nepal Demographic Health Survey (NDHS) of 2011 reported that one in five (22%) women aged between 15 and 49 years has experienced physical violence at least once in their lifetime, and 12% have experienced sexual violence perpetrated by their husband. The same report cites mothers-in-law and fathers-in-law as perpetrators of physical violence (4).

According to the WHO multicountry study on Women's Health and Domestic Violence against Women, DV during pregnancy ranges from 1 to 28% (3). In Nepal, 6% of women experience DV during pregnancy (4).

DV during pregnancy has detrimental effects on the health of the mother and her newborn. Postpartum depression, anxiety, and post-traumatic stress disorders are common negative maternal mental health consequences (5). In addition, perinatal and postnatal mortality, and low birth weights are some of the negative consequences among the newborns (6–9).

Health care providers (HCPs) such as doctors, nurses, and auxiliary nurse midwives play an important role in addressing DV because victims may present to health care with physical injuries and psychological problems that need treatment (1, 10). HCPs providing antenatal care (ANC) are in a unique position to create a safe and confidential environment to facilitate disclosure of violence and offer appropriate support services (3). Moreover, in Nepal, ANC could provide a ‘window of opportunity’ because the NDHS reports that 71% of the women visit health centers (HCs) four or more times during their pregnancy, and 15% women visit the HCs at least once. In addition, the survivors seek help from the HCs twice as often than legal services, suggesting that the HCPs are still the first point of contact (4).

Despite this, most of the women do not disclose their experience of DV at the HCs because of reasons such as normalization of DV; saving the family prestige, love, and affection toward their husband; fear of breaking up the family; fear of threats from the perpetrators; and lack of awareness about formal support services (11, 12). Wijma et al., in their case study, reported that concealing violence nurtured anxiety, guilt, and shame, and it is ‘silenced’ further. If the HCPs listen to the women, they might reveal their story, which might help break the cycle of abuse (13). Thus, identifying and assisting women who have experienced DV in time through the ANC may offer improved services and a better life for the survivors (14, 15).

In Nepal, interventions like One Stop Crisis Management Centers (OCMCs) have been established to assist these women (16). However, women have not been asked about what their needs are from the HCs. Therefore, the aim of our study was to explore how the Nepali women evaluate their ANC and their expectations of the ANC in relation to DV during pregnancy.

## Methods

### Study design

This qualitative study attempts to understand the world from the subjects’ point of view, to unfold the meaning of their experiences prior to scientific explanations (17). To get a better understanding and garner the experiences of these women, in-depth interviews were conducted.

### Study setting

Nepal is a landlocked country in between China and India with 26.4 million inhabitants, of which 1.7 million live in Kathmandu. Nepali is the common language spoken by the inhabitants. Sixty-seven percent of men and 58% of women are literate. Women residing in urban area are more educated than women in the rural area. Although the status of Nepali women is improving, the gender-related development index is still low (0.499) (18).

### Participants’ characteristics

The women in our study were between 22 and 45 years of age. The majority of them got married at an early age (<16 years). They had 2–5 children. Most of them had migrated from the rural parts of Nepal but had been living in Kathmandu and Bhaktapur for a long time (Table 1).

**Table 1:** Socio-demographic characteristics of the study participants (*N*=12)

Characteristics	*n*
Age (years)	
20–30	5
30–40	6
>40	1
Educational status	
No education	6
Did not complete high school	1
Completed high school	3
Graduate or higher	2
Employment status	
Currently employed	8
Unemployed	4
Age at marriage (years)	
≤16	6
>16	6
Number of ANC	
<4	4
>4	8
Acts of DV experienced during pregnancy	
Beating; hitting	8
Insults; accusation (seeing another man, being a thief, sleeping with husband's friend, not having children); use of obscene language	12
Sexual violence	3
Infidelity	3
Dowry-related violence	1
Neglect; made to do heavy work	4
Economic violence	5
Perpetrators of DV	
Husband	11
Ex-husband	3
Mother in law	5
Father in law	2
Other in laws	4

Physical, psychological, and sexual violence by husband and mother-in-law were the most common forms of DV experienced by women (Tables 1 and 2).

**Table 2:** Experiences of domestic violence by women

‘… he [my husband] was a drug addict … he did not earn [money] … we had nothing to eat. We had to sleep on the floor … we had only one mattress. He would be away many days, and he came home drunk. He always had his friend at home … we [I, my husband and his friend] had to share the same mattress. His friend forced me for that [to have sex with him]. The next morning, my husband scolded me and beat me because I slept with his friend … One day, I got pregnant with his [husband's] child. His friends would insult me and say that even they [his friends] have share in my baby … I felt very bad [crying]. One day my husband came home after many days … drunk. I asked for money [to pay the rent] … he asked me for food. I had not cooked anything [because I had nothing at home] … I had not eaten for many days … and we started fighting. He hit me very hard and pushed me. I fell on the basin full of water and pots and pans. I started bleeding … he was afraid that I was bleeding and took me to the hospital’. [Interview 3]

### Data collection

We included women purposively according to socio-demographic characteristics, experiences of DV, and number of ANC visits in collaboration with two NGOs: Women's Rehabilitation Center (WOREC) and Community Action Center, Nepal (CAC Nepal) in Kathmandu and Bhaktapur, respectively. WOREC's work is related to gender-based violence, in particular DV. The organization runs seven safe houses in many regions across Nepal. Similarly, CAC Nepal has some of their activities focused on DV. They have crisis centers for the victims of DV and one drop-in-center in Bhaktapur.

Due to different organizational structure at these two NGOs, the process of recruitment of the women differed. At WOREC, women were identified from the case registers by the first author (PR) with the help of safe house in-charge. The safe house in-charge phoned the women for the interview. Whereas at CAC Nepal, co-workers identified the women for the interview during community visits and scheduled the interviews. Some of the women were still living in an abusive situation, others were not living with their husband or their family, and some had been re-integrated in their family with the help of the NGOs.

Moreover, the initial eligibility criteria were to recruit women who had experienced DV and had attended four ANC visits. However, the last criteria had to be revised as only a few eligible women had attended at least four ANCs as per the national safe motherhood program (19). Therefore, we adjusted the eligibility criteria to include women who had been to ANC at least once.

In total, 14 women were approached for the purpose of our study. Two women did not consent to participate mainly because they had already been re-integrated into the family and did not wish to recall their experience.

A topic guide was developed and finalized by the authors, which was translated and pretested before the first interview (Table 3). The first author (PR) conducted 12 audio-recorded in-depth interviews in Nepali. Written notes were taken following consent from the participants regarding their non-verbal communication. The interview began with introduction to build rapport before the women were guided to share their experience of DV during pregnancy, evaluation of their ANC visit, and their needs and expectation from ANC (Table 3). The duration of the interview ranged from 35 minutes to 1 hour.

**Table 3:** Interview guide for in-depth interviews

1.	Introduction
2.	*Marriage/relationship with husband*. Probing questions for follow-up information: occupation of the husband, how they met, married life, violence (insulted, shouted, threatened, hit, kicked, slapped, forced to sexual activity)
3.	*Relationship with the in-laws*. Probing questions about violence, ask precise questions about the violence [type, severity, frequency?]
4.	*Pregnancy*. Probing questions: how they felt [physical and emotional health?], place of delivery, antenatal checkups, violence during pregnancy
5.	*Disclosure about experience(s) of violence*. Probing questions: who knew about the violence? Whom did you tell? Did you tell anyone at the health center? How did they respond? Their expectations from the antenatal care providers
6.	Recommendations to help improve the quality of care for women who have experienced domestic violence during pregnancy and who come for antenatal care

### Data analysis

The interviews were transcribed verbatim in Nepali and then translated into English by the first author (PR) for the analysis. The second author (SKJ), who is also from Nepal, read the verbatim transcriptions to increase trustworthiness and ensure conformity of the translation.

The analysis then followed the steps of Graneheim and Lundman content analysis (20). At first, the interviews were read through several times to obtain a sense of the whole incorporating non-verbal communication noted during the interviews.

Then, the meaning units were identified from the interviews by the first author. Graneheim and Lundman consider meaning unit as words or paragraph containing aspects related to each other through their content (Table 4 illustrates the analyzing process). Thereafter, the meaning units were condensed, which Graneheim and Lundman refer to as a process of shortening of meaning units while still preserving the core. Finally, the condensed meaning units were abstracted and labeled with codes. All the authors reflected upon the meaning units, condensed meaning units, and discussed the different codes. Subsequently, the codes were grouped according to their similarities and differences into subcategories. These subcategories were grouped into three categories. Thereafter, the tentative subcategories and categories were discussed among all the authors. During this process, reflection on the interviews, subcategories, and categories continued until no new subcategories and categories emerged (Table 4).

**Table 4:** Example of content analysis

Meaning units	Condensed meaning units	Codes	Subcategories	Categories
No, I would not have told them (about abuse). I told you because you seem to understand my pain and you also want to get information from me. Others will think that I am useless and they start judging me, don't they? One has to understand, what pain people can experience, how a person's fate will be	If one does not understand the pain and starts judging women, you do not want to disclose	Judgment as a barrier to disclosure	Barrier to disclosure of domestic violence	
There (hospital) health care provider's started telling in the emergency that my case might be a police case if I would tell them that I had been beaten, so I told them that I fell from the ladder while I was carrying a tub and to call the doctor. I felt that if it would been a police case, I would be left alone and I would not have any place to go on top of that I was pregnant, (crying) so I did not tell anyone	Did not disclose and lied about the violence due to fear of being alone if it would become a police case	Concealment of domestic violence due to fear of being alone during the pregnancy	Concealment of domestic violence	Enduring domestic violence – a hidden burden

The analysis process continued until an agreement was reached on each of the subcategories and categories among all the authors, challenging each other's understanding based on personal and professional backgrounds. All the authors have knowledge of qualitative methods and experience of working in national and international health care systems and researching DV. All authors discussed and agreed upon the final subcategories and categories (Table 4). Authentic citations are given to support and confirm the findings and represent reality.

## Ethical considerations and ethical clearance

The ethical guidelines recommended by WHO was followed when the study protocol was developed and throughout the study (21–23). To ensure the women's autonomy, women were informed about the objective, the risks, and the benefits of the study, as well as the notion of voluntary participation. The women were given the opportunity to decline or reschedule the interview or withdraw from the interview whenever if needed. Verbal informed consent was obtained. To ensure the women's safety, the identity of the women was kept anonymous.

Ethical approval to conduct the study was obtained from the Norwegian Research Council and the Nepal Health Research Council. The executive directors of WOREC and CAC, respectively, granted their verbal permission to conduct the interviews.

## Results

Three main categories emerged from the in-depth interviews and are discussed as follows: 1) enduring domestic violence – a hidden burden, 2) all we need is an opportunity, and 3) made a bad thing worse (Table 5).

**Table 5:** How women with domestic violence evaluate their antenatal care?

1.	Enduring domestic violence – a hidden burden
•	Concealment
•	Barriers to disclosure
2.	All we need is an opportunity
•	Routine enquiry
•	Support focused on women
•	Other support for family members
3.	Making a bad thing worse
•	Neglect
•	Emotional abuse
•	Physical abuse

### Enduring domestic violence – a hidden burden

The women in our study concealed incidences of DV and lived within their private sphere, enduring the burden. During the interviews, the women described reasons for concealing DV during pregnancy.

#### Concealment

Fear was one of the main reasons for why women concealed being victims of DV. The women had fear of being insulted, discriminated against themselves or towards their family, being re-victimized by the husband, and also being left alone during their pregnancy. One woman said:

… They [HCPs] said it might be a police case, and if I said that I had been beaten by my husband, they would call the police. I would be left alone. I would not have any place to go [crying]. I lied and told them that I fell from the ladder while I was carrying a tub… I did not tell anyone. [Interview 3]

Similarly, another woman said that even though she stayed at the hospital, she did not tell anyone about her bruises because of fear of being thrown out of the house. Victim blaming, guilt, being insulted by others in the community, shame, and shyness were other reasons given by the women for concealing DV.

#### Barriers to disclosure

Meeting a professional HCP did not evoke feelings of trust where the women could disclose their experiences. One of the women explained that she did not tell the HCP about DV during pregnancy because she felt that the HCPs might laugh at her. Another woman shared concerns that the HCPs might have thought that she was useless and judge her as not being a good wife. One of the women said, ‘I will never tell a health worker especially nurses because they have their own way [of dealing], they just walk away’.

The majority of the women felt that the HCPs showed no empathy toward them. One woman emphasized, ‘They [HCPs] should not talk to us like that [rudely]’. Another woman said that if HCPs asked open questions like, ‘What happened to you?’ it would encourage them to share their experiences. The women did not disclose experiences of DV because they were not asked, or when asked, they had to face attitudes like insensitivity, blame, and judgment. Moreover, they felt that there was ‘no advantage [to share information about having been victims of DV]’:

… A woman [I knew] disclosed to the HCPs about being abused [raped] and that she did not want the baby because the child had no father. The HCPs were afraid that she would run away from the hospital without paying the bill and did not let her go. Nobody sought a solution. That is why I never say anything. [Interview 3]

### All we need is an opportunity

Women during the interview shared what they wished from the HCPs. The women wished for an opportunity to share their experience with them. They stated that the HCPs should enquire about their experience of DV. The woman also suggested compassionate, appropriate, individualized care from the HCPs.

#### Routine enquiry

One of the women said that if the HCPs asked them about DV, it would give them an opportunity to share their experience with them and get advice for them and their baby. Women wished that HCPs understood their pain. As one of them said, ‘I told you [interviewer] because you seem to understand my pain’. Women said that empathy, politeness, love, and respect would motivate them to share their experience of DV. ‘… They [HCPs] should ask about what had happened to us, how we got to the hospital, who abused us’, said one of the women.

Women also expressed that it was important that the HCPs maintain privacy and confidentiality when they asked about DV. They added that if the HCPs guaranteed not to tell anyone, they would be more willing to share their experiences. ‘I expect you will not tell anyone’, said one woman to the interviewer, making clear the need for confidentiality.

Furthermore, one of the women felt sorry for the HCPs and stated, ‘maybe the HCPs do not know what to do’. So the woman suggested that training might help the HCPs: ‘… We cannot expect that they [HCPs] will be able to ask and manage without any training on DV during pregnancy’.

#### Support focused on women

Women held different views on what the HCs could provide as a means of support. Some suggested that the HCPs should refer women who have experienced DV to the HCs that provide services free of charge to poor women. In contrast, others accepted that a nominal fee would be good when they have to do investigations like blood test and ultrasonography.

… women like us [who have been abused] die to see 1–2 Rupees [Nepali currency]. It would be nice if we get services according to how much money we have. If hospital could give us services at a discount, it's fine. [Interview 5]

Most of the women visited the public maternity hospital for their ANC because it is free of charge. Thus, the women suggested the need for another specialized hospital, one that was especially focused on women who have experienced DV. ‘Nowadays, we have reserved seats for women and separate bus for women. Like that, we should have a special unit for pregnant women living with DV’. Similarly, a few women stated that awareness about HCs that provide facilities for women in relation to DV was important because most of the women did not know about such services. They said, ‘No one will use the services if they do not know about it’. In addition, one woman emphasized that a female HCP would understand them better than a male HCP, she said, ‘A woman will understand another woman's problem’.

#### Other support for family members

Apart from the services focused on them, the women also expected supports for the perpetrators. A woman stated that rehabilitation of her husband who is an alcoholic would help to reduce the DV she experienced. Others expressed that it was very important to educate couples and families about DV. They also wished that the HCPs created an environment to talk to the family members and counsel them in various issues such as nutrition during pregnancy, danger signs during pregnancy, and indirectly about DV and the legal aspects of DV.

Even when my doctor did my checkup, he did not say anything [follow-up, diet during pregnancy] or ask me to bring anyone from my family … If the doctors could convince the family members about the care needed during pregnancy, they could help women like us … My mother-in-law said that she delivered her baby without eating fruits and doing strenuous work. She said I was acting smart. [Interview 1]

### Making a bad thing worse

During the interviews, most of the women expressed examples of the types of negative behavior exhibited by the HCPs. Most of them described it as ‘bad behaviors’.

#### Neglect

A few women described ‘*neglect*’ as a failure to care for women who have experienced DV by the HCP's. The women gave some examples of failure to care. One woman disclosed that there was no medicine and no HCPs when she visited the ANC. Others felt that the HCPs did not care for them because they had very little time for the women. ‘What the HCPs try to do is to finish their work and go home. The system is like that and therefore women do not like to go there’. Women also felt that the HCPs did not inform and advise them adequately, concerning follow-up and medication during pregnancy. One woman also stated that the HCPs did her routine ANC but she did not check for or look at the bruises that she had on her body.

#### Emotional abuse

Expressions such as ‘… they talked loudly and rudely’ and ‘… They scold us’ were shared by the women to describe emotional abuse. Similarly, one woman said that she had gone to the hospital because she wanted to ask when it was best to plan for a baby. She told the doctor that her husband forcefully had sex with her, and she asked the doctor if he could talk to her husband and convince him to stop forcing her. She was unsatisfied with the doctor's insensitive response.

… Then he told me that *I* should talk to my husband and it was my responsibility to convince my husband. He used words like *maybe you also need sex*. I felt very bad because he talked to me like that. He was a male doctor. [Interview 8]

#### Physical abuse

Some women reported incidents of physical abuse during labor. One woman said that the nurses pressed on her abdomen to listen to the baby's heartbeat, and when she asked the nurse not to press the abdomen, the nurse replied, ‘You should bear the pain’. Another woman described how a nurse treated her when she went to the hospital for her delivery.

The nurse beat me … one shouts, one cries during labor, but the nurse slapped me on my thigh. I cried because I was in pain. I had even told them that I did not have anyone to [support me]. They should have treated me well. They should have behaved well and not hit me. [Interview 4]

## Discussion

Women in our study had experienced diverse forms of DV during pregnancy. However, they concealed their experiences from health workers and lived with the burden because they were afraid of being abandoned during their pregnancy. The women felt that HCPs humiliated them. Some women also felt that HCPs were insensitive and discriminated them. On the contrary, the women wished that the HCPs would enquire about DV and show empathy toward them.

In Nepal, women are expected to tolerate DV for the sake of their family (11). Earlier studies have shown that women hesitated to reveal their experiences because they were embarrassed or afraid to disclose about DV themselves; they feared public scrutiny, being stigmatized as victims, and attitudes and response of the HCPs to DV. Judgmental and insensitive attitudes of HCPs toward them were barriers to disclosure (24–26).

Professional ethics and women-centered care advocate that women should be provided with practical care and support that responds to her concern, ensuring her privacy and confidentiality to reduce further harm (3). However, women in our study shared that the HCPs shouted, scolded, and slapped them. Globally, studies have documented disrespect, beating, verbal abuse, and mistreatment of women during childbirth, feeling powerless, being ignored, lack of emotional support, and callousness as abuse in health care (27–30). Nevertheless, HCPs do not perceive their behavior toward these women as abusive. Some HCPs consider abuse in health care as ‘small things’, but most of them express it as ‘innocent thoughtlessness’ (31, 32). Moreover, if the HCPs abuse women who have experienced DV during pregnancy, women lose their trust in them. As a result, they utilize the ANC less, which means a window of opportunity is lost, hindering disclosure of DV and increasing the risk for complications in pregnancy and childbirth.

The WHO has defined social well-being as one of the important components of health (33). Although HCPs cannot ensure social well-being directly, they can create a non-judgmental, respectful, and compassionate environment to motivate women to disclose their experience of DV and contribute to reducing harm caused by DV (10, 34, 35). Women in our study preferred being asked about their experiences by the HCPs similar to the findings from the earlier studies (36). Routine inquiry alone may not be helpful. However, a non-judgmental way of enquiring may uncover the hidden experience of DV among women and trigger the need to seek help earlier or at later stage from ANC, which ultimately increases women's safety because initially many women may not even recognize themselves as being victims of DV (15, 37, 38).

In addition, the women in our study emphasized the importance of privacy and confidentiality in their interactions with the HCPs and the ANCs. Earlier studies have also shown that women expect that the HCPs would ensure their privacy and confidentiality (39). However, in Nepal, ensuring privacy and confidentiality at the ANC could be challenging because of the infrastructure. The ANC clinics are busy and crowded, where the pregnant women are examined in cubicles partially partitioned with curtains or walls.

There is a need for transformation in the attitudes of the HCPs toward DV. How can a HCP who agrees to wife beating by the husband care for women? An effective training may enable HCPs to respond appropriately to the needs of the women, and challenge the issues of power and abuse and mitigate the concerns about opening a ‘Pandora's Box’ (34, 40). Mainstreaming gender, DV, and its management within ANC into the curriculum in medical and nursing schools could help the HCPs to identify and assist survivors of DV.

Quality of care for survivors of DV is a multidimensional and multisectorial challenge because of its complexity. The HCs alone cannot achieve the goal although they are crucial gateways to specific services (eg. treatment of phyiscal injuries), and referral to other services should be mandatory if required (3). However, an assessment of the performance of hospital-based OCMCs in Nepal reports that even the medical staffs have not been oriented about OCMCs provided by the hospitals that they work at (16). Moreover, to improve ANC programs, it is necessary to understand the providers’ perspective as well (41). Thus, there is a need to explore the perceptions of DV and ANC and abuse in health care from the HCPs’ perspective.

Help seeking among survivors of DV is a dialectical process where a woman defines her problem first, and then decides to seek help and selects a source (formal and informal) for help (42). The survivor is most receptive to interventions only when she realizes that she is a victim, and that is when she is likely to reach out for help (43). A formal source like the ANC could offer assistance to prevent and reduce DV and provide alternative support. (35). Some of the women in our study concealed DV and chose to live with a husband who abused them. Therefore, an intervention to promote safety of the women could help reduce harm to the mother and the newborn. A study conducted in the United States has shown that if women are aware of safety-promoting behavior, it could prevent women from further harm due to DV (44). Therefore, there is a need to replicate the study in Nepal and assess the feasibility of teaching safety-promoting behavior from ANCs. In addition, some of the women in our study expressed that education of other family members about nutrition, health, DV, and law to protect victims of DV could prevent DV during pregnancy. Therefore, integration of women's health education, DV during pregnancy, and legal issues of DV through ANC for the husband, mother-in-law, father-in-law, and other in-law members could help reduce DV during pregnancy.

In Nepal, DV has been recognized as a public health concern, in particular for safe motherhood and women's health since 2002 (45). Recently, a clinical protocol for gender-based violence (GBV) has been published by UNFPA with instructions on examination, reporting of violence, and counseling and supporting women; however, the protocol does not include guidelines to manage DV during pregnancy. It is applicable for women who are brought to the OCMCs by organizations or police and does not apply to women who do not self-report DV. Moreover, only a few women are aware of formal services such as OCMCs (16). Apart from this, there is a need of standard protocol that guides HCPs on how to identify women about DV.

### Strengths and limitations

This is the first study performed in Nepal to evaluate the experience and expectation of utilizing ANC among women survivors of DV during pregnancy. Furthermore, our explorative in-depth interviews have generated new knowledge regarding abuse in health care.

Through purposeful recruitment of women, we have captured diverse experiences in regard to the study aim based on women's different socio-demographic background as well as various experiences of DV. The interviews were conducted in Nepali, in a secluded room which ensured confidentiality and helped to build trust among the women. This contributed to a comfortable and open dialogue between the interviewer and the women. It is our opinion that all the above-mentioned measures helped us to gather rich material. However, we could have built a higher level of trust if we had been able to meet these women on multiple occasions to garner an in-depth understanding. To increase the trustworthiness of our findings, we have described all procedures used in the study as thoroughly as possible (46, 47).

As with all translations in cross-language research, it was difficult to retain the conceptual similarity of the proverbs and phrases used in local language. However, the first author is fluent in both Nepali and English, so the context of the interview and meaning of local phrases were thoroughly described while translating the interviews to ensure linguistic nuances such that the other authors reading only the English translations could interpret and reflect on the interview during analysis. Including multiple researchers in the analysis also increases the credibility of the findings of our study.

However, our study includes self-reported DV and women living in the urban area. Thus, transferability of our findings is limited and may not represent perception of women living in the rural areas. A random selection of women from both the urban and rural communities could have broadened the understanding.

## Conclusion

Our study has broadened the understanding of how women who have experienced DV evaluate their ANC; it also allows us to understand what their expectations are from the ANC and the HCPs. Furthermore, our study has generated findings on abuse in health care, which has been perceived as barriers to disclosure of DV by some women. Enquiries made by the HCPs may provide an opportunity for the women to seek help at some point in their life. Interventions to promote use of safety-promoting behavior through the ANC could be a step forward in preventing women and their newborns due to DV during pregnancy.

## Paper context

Domestic violence during pregnancy has detrimental effects on the health of the mother and her newborn. Antenatal care could provide a window of opportunity to identify and assist those who suffer domestic violence. Our research provides women's perception of antenatal care as a sufferer of domestic violence during pregnancy and their expectation from antenatal care. Findings from this study could be an eye opener to health care providers and may help in strengthening services for these women in Nepal.
